# Transcriptome analysis revealed potential mechanisms of channel catfish growth advantage over blue catfish in a tank culture environment

**DOI:** 10.3389/fgene.2024.1341555

**Published:** 2024-04-29

**Authors:** Haolong Wang, Baofeng Su, Ying Zhang, Mei Shang, Jinhai Wang, Andrew Johnson, Hamza Dilawar, Timothy J. Bruce, Rex A. Dunham, Xu Wang

**Affiliations:** ^1^ Department of Pathobiology, College of Veterinary Medicine, Auburn University, Auburn, AL, United States; ^2^ Auburn University Center for Advanced Science, Innovation, and Commerce, Alabama Agricultural Experiment Station, Auburn, AL, United States; ^3^ School of Fisheries, Aquaculture and Aquatic Sciences, Auburn University, Auburn, AL, United States; ^4^ Scott-Ritchey Research Center, College of Veterinary Medicine, Auburn University, Auburn, AL, United States; ^5^ HudsonAlpha Institute for Biotechnology, Huntsville, AL, United States

**Keywords:** aquaculture, catfish growth phenotype, tissue, organ, transcriptome, expression marker gene

## Abstract

Channel catfish (*Ictalurus punctatus*) and blue catfish (*Ictalurus furcatus*) are two economically important freshwater aquaculture species in the United States, with channel catfish contributing to nearly half of the country’s aquaculture production. While differences in economic traits such as growth rate and disease resistance have been noted, the extent of transcriptomic variance across various tissues between these species remains largely unexplored. The hybridization of female channel catfish with male blue catfish has led to the development of superior hybrid catfish breeds that exhibit enhanced growth rates and improved disease resistance, which dominate more than half of the total US catfish production. While hybrid catfish have significant growth advantages in earthen ponds, channel catfish were reported to grow faster in tank culture environments. In this study, we confirmed channel fish’s superiority in growth over blue catfish in 60-L tanks at 10.8 months of age (30.3 g and 11.6 g in this study, respectively; *p* < 0.001). In addition, we conducted RNA sequencing experiments and established transcriptomic resources for the heart, liver, intestine, mucus, and muscle of both species. The number of expressed genes varied across tissues, ranging from 5,036 in the muscle to over 20,000 in the mucus. Gene Ontology analysis has revealed the functional specificity of differentially expressed genes within their respective tissues, with significant pathway enrichment in metabolic pathways, immune activity, and stress responses. Noteworthy tissue-specific marker genes, including *lrrc10*, *fabp2*, *myog*, *pth1a*, *hspa9*, *cyp21a2*, *agt*, and *ngtb*, have been identified. This transcriptome resource is poised to support future investigations into the molecular mechanisms underlying environment-dependent heterosis and advance genetic breeding efforts of hybrid catfish.

## Introduction

Channel catfish (*Ictalurus punctatus*) and blue catfish (*Ictalurus furcatus*) are two native North American catfish species. Blue catfish are the largest catfish in the US and reach sexual maturity at older ages than channel catfish (Graham, 1999). Blue catfish grow slower than channel catfish during the first 2 years. As a result, channel catfish reach market size before blue catfish. Channel catfish is traditionally considered the most important and popular species for catfish farmers and producers in the US. However, channel catfish is not as resistant as blue catfish to enteric septicemia of catfish (ESC), which causes an annual economic loss of 50 million dollars (Wolters and Johnson, 1994; Yeh et al., 2005).

To combat pathogenic infections in channel catfish, genetic enhancement of catfish was achieved through interspecific hybridization. The hybrid between female channel catfish and male blue catfish (*I*. *punctatus* × *I*. *furcatus*) constitutes more than 50% of the total US catfish production (Torrans and Ott, 2018), and the hybrid catfish grow 20%–100% faster than commonly cultured strains of channel catfish, depending upon the environment (Dunham et al., 1990; Dunham et al., 2008). The hybrid catfish combines many of the best traits of their parental species and is a highly desirable fish in commercial pond culture. It offers improved feed conversion efficiency (Dunham et al., 2008; Brown et al., 2011), increased carcass yield (Bosworth, 2012), better low oxygen tolerance (Dunham et al., 1983), disease resistance (Arias et al., 2012), and enhanced harvestability (Dunham and Masser, 2012). However, heterosis in growth is environment-dependent. Catfish fry are typically reared in indoor tanks, and juvenile catfish will be transferred from indoor tanks to earthen ponds for aquaculture. Previous studies on the growth trait have shown that hybrid catfish are not superior in the tank culture environment, whereas channel catfish had a growth advantage instead (Dunham et al., 1990). The molecular mechanism of growth advantage in channel catfish is still poorly understood. Gene expression research sheds light on the physiology of a set of cells or tissues at a certain period, including cellular adaptations to different environments (Singh et al., 2018). Although organism cells carry out similar processes for key biological functions in their own tissue environment, they display unique functions that support the definition of their phenotype (Sonawane et al., 2017). Characterizing gene expression differences between the two catfish species across various tissues will provide valuable insights into understanding the phenotypic variations in growth and disease resistance and serve as the first pass to investigate molecular mechanisms underlying environment-dependent heterosis.

RNA sequencing is a transcriptome-wide approach used to characterize gene expression profiles in various catfish species. Several studies have recently been conducted to analyze differentially expressed genes (DEGs) and functional pathways in blue catfish, channel catfish, and their hybrids. For instance, in the liver transcriptome, a group of genes associated with fatty acid metabolism was discovered to be significantly upregulated in channel catfish compared to blue catfish and hybrids (Wang et al., 2022a). In another study, a set of DEGs involved in the formation of the swim bladder were identified between channel catfish and other catfish. These genes were enriched in the Wnt signaling pathway and the hedgehog signaling pathway (Yang et al., 2018). Taking advantage of RNA sequencing, these findings shed light on the distinctive genetic characteristics and potential functional differences between these catfish species and their hybrids. However, a broader organ selection is still needed to better understand the transcriptomes in channel catfish and blue catfish.

In this study, five organs (heart, liver, muscle, mucus, and intestine) were selected from channel catfish and blue catfish transcriptome characterization at the 10.8-month juvenile fish stage. Muscle development and muscle growth are directly relevant to the overall quality of fish meat (Fuentes et al., 2013; Xu et al., 2019). The heart plays a crucial role in pumping oxygen and nutrients and dealing with environmental stress (Saetan et al., 2021). The liver is involved in various metabolic processes that support fish growth, including but not limited to metabolism, nutrient storage, energy production, and detoxification (Zhang et al., 2021). Mucus serves as a protective layer against pathogens, parasites, and environmental toxins, as well as a barrier to fight infection through its immune-related functions (Lange et al., 2018). The intestine also has an immune function to defend against harmful microbes and interact with the gut microbiota. In addition, it is the major organ for digestion and nutrient assimilation, which promotes growth. The comparative transcriptomic analyses provide insights into gene function differences between the two species and the molecular basis of the channel catfish’s growth advantage in the tank culture environment.

## Materials and methods

### Fish maintenance and tissue sample collection

The experimental animal protocols regarding animal care and tissue collections were approved by the Auburn University Institutional Animal Care and Use Committee (AU-IACUC) with the approval number PRN-2019-3520. Blue catfish (BB) and channel catfish (CC) were cultured at the Auburn University Fish Genetics Research Unit in Auburn, Alabama, United States. Both catfish species were maintained in the indoor recirculatory aquaculture system with separate 60-L rectangular tanks (60 cm × 23 cm × 43.5 cm) at an initial density of 1,000 fry per tank. The fry were fed with Purina^®^ AquaMax^®^ Fry Starter 100 for the first 3 months. At 2 months old, fry density was adjusted to 100 fry per tank and then to 50 fish per tank at 4 months old. Starting at 4 months, the fry were fed with Purina^®^ AquaMax^®^ Fry Starter 200 for 3 months and then with Purina^®^ AquaMax^®^ Fry Starter 300 three times a day. Dissolved oxygen was maintained above 5 mg/L, with pH levels between 7.0 and 7.5. At 10.8 months of age, three randomly selected fish from each species were euthanized with 300 ppm tricaine methanesulfonate (MS-222, Syndel Inc., Ferndale, WA, United States). Muscle, liver, intestine, mucus, and heart tissues were dissected immediately after euthanasia. All tissue samples were flash-frozen in liquid nitrogen and stored in a −80°C freezer until RNA extraction.

### RNA extraction, library preparation, and sequencing

Three replicates were performed for each tissue of the two catfish species at 10.8 months of age. The total RNA was extracted using the Quick RNA Microprep Kit (Zymo Research, Irvine, CA, United States) following the manufacturer’s protocol. RNA concentrations were measured using a NanoDrop One^C^ Microvolume UV-Vis Spectrophotometer (Thermo Scientific, Waltham, MA, United States). The library for each tissue sample was constructed using the NEBNext^®^ Poly(A) mRNA Magnetic Isolation Module and NEBNext^®^ Ultra™ II RNA Library Prep Kit (New England BioLabs, Ipswich, MA, United States) with 1 µg of total RNA input. The library PCR amplifications were conducted using 18 cycles. The concentration of sequencing libraries was quantified using a Qubit 3.0 Fluorometer (Thermo Scientific, Waltham, MA, United States), and the average size of cDNA libraries was evaluated with the D1000 ScreenTape assay using the TapeStation 4,200 System (Agilent Technologies, Santa Clara, CA, United States). The libraries were sequenced on an Illumina NovoSeq6000 sequencer to generate 2 × 150-bp paired-end reads at Novogene (Novogene Corporation Inc., Sacramento, CA, United States).

### RNA sequencing analysis

The quality of the raw reads was assessed by FastQC (version 0.11.6) (Andrews, 2010). Low-quality bases and adapter sequences were trimmed using Trimmomatic (version 0.39), and sequencing reads shorter than 36 bp in length were excluded from subsequent analysis (Bolger et al., 2014). RNA-seq reads were aligned to the blue catfish (*I. furcatus*) reference genome (Wang et al., 2022b) using STAR aligner version 2.7.5c (Dobin et al., 2013). The gene read counts of each sample were quantified and summarized using HTseq version 1.0 (Anders et al., 2015). Genes with extremely low expression values in all tissues were excluded, and genes with counts >1 in at least three samples were retained for subsequent analysis.

### Identification of differentially expressed genes

To determine gene expression levels for each sample, read counts were normalized using the edgeR package in R (version 3.6.4) (Robinson et al., 2010). The differentially expressed genes (DEGs) between CC and BB for each tissue sample were identified using the cutoff of |log2 (fold change)| > 1.5 and a false discovery rate (FDR) < 0.05. The Benjamini–Hochberg method was used to determine the adjusted *p*-values.

### Gene ontology and functional enrichment analysis

For functional enrichment analysis, blue catfish genes were mapped to the zebrafish (*Danio rerio*) assembly GRCz11 (Howe et al., 2013) using DIAMOND version 2.0.0 (Buchfink et al., 2021) to determine the gene names. Gene ontology (GO) and Kyoto Encyclopedia of Genes and Genomes (KEGG) pathway (Kanehisa and Goto, 2000) analyses were performed using Metascape (Zhou et al., 2019) with default parameters. The DEG gene symbols were used as the input gene list. The optional parameters of “input as species” and “analysis as species” were selected as “any species” and “zebrafish,” respectively. The GO terms analysis was conducted for biological processes, cellular components, and molecular functions.

### Analysis of tissue-specific gene expression

The τ index was used to determine the tissue specificity (Yanai et al., 2005) in gene expression, which ranges from 0 (non-specific, expressed equally in all tissues) to 1 (highly specific, only expressed in one tissue). For each gene, τ is computed according to the formula τ index = $\frac{\Sigma_{i}^{N}\left(1-x_{i}\right)}{N-1},i=1$ , where *N* is the number of tissues, and *x*_*i*_ is the normalized expression value. A cutoff of τ > 0.9 was used to detect tissue-specific genes (Yanai et al., 2005).

### Quantitative reverse transcription PCR validation of tissue-specific genes

Quantitative reverse transcription PCR (qRT-PCR) experiments were performed to validate the tissue-specific genes identified from RNA-seq data. One candidate gene from each group (channel catfish only, blue catfish only, and both species) was selected, including *fabp2*, *cyp21a2*, and *pth1a*. A housekeeping gene, *gapdh*, was included as a reference. The relative gene expression levels of these genes were quantified in the heart, intestine, liver, mucus, and muscle of channel catfish and blue catfish, with three replicates for each tissue. Primer sequences used for qRT-PCR validation are listed in Supplementary Table S4. The first-strand cDNA synthesis was conducted using the LunaScript^®^ RT SuperMix Kit (New England BioLabs, Ipswich, MA, United States) with 1 μg of total RNA, following the manufacturer’s protocol. The same total RNA samples for the RNA-seq experiments were used for validation. Quantitative reverse transcription PCR was performed on a Bio-Rad C1000 Touch Thermal Cycler with CFX96 Real-Time PCR Detection Systems (Bio-Rad Laboratories, Hercules, CA, United States) in a 20 μL final reaction volume. The reaction mixture included 10 μL of Luna universal qPCR Master Mix, 0.5 μL of each primer, 6 μL of nuclease-free water, and 3 μL of cDNA template. The standard amplification protocol was 95°C for 60 s, followed by 40 cycles at 95°C for 15 s and 60°C for 30 s with two technical replicates. The relative gene expression value was computed using the 2^−ΔΔCT^ method.

## Results

### Channel catfish exhibit superior growth during the early life stages of tank culture

**FIGURE 1:**
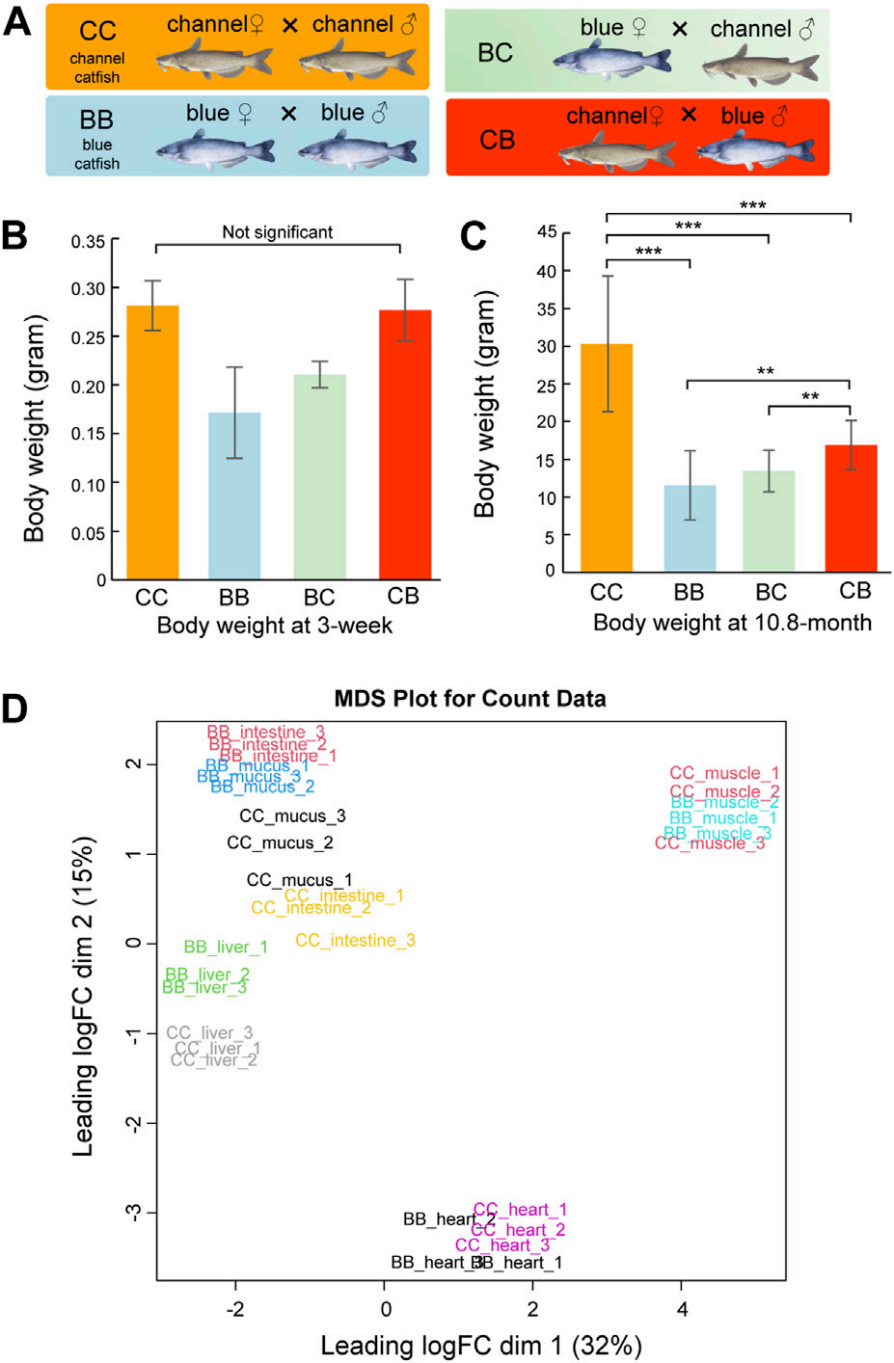
Body weight measurements and multi-dimensional scaling (MDS) plot of transcriptome quantification from five tissues of channel catfish and blue catfish. **(A)** Four genetic types of channel catfish *Ictalurus punctatus* (CC), blue catfish *Ictalurus furcatus* (BB), and their reciprocal hybrids (BC and CB). **(B)** Barplot of body weight for four genetic types at 3 weeks of age. **(C)** Barplot of body weight for four genetic types at 10.8 months of age. Statistical significance was assessed by the nonparametric Mann–Whitney *U* test (**, *p* < 0.01; ***, *p* < 0.001). **(D)** MDS plot of five tissues in blue catfish (BB) and channel catfish (CC). The expressed gene counts were used as input. The *x*-axis and *y*-axis represent the first two dimensions.To graphics: This image should be moved to figure 1

Although heterosis has been reported in pond culture, channel catfish (CC, channel catfish × channel catfish) exhibit superior growth compared to blue catfish (BB, blue catfish × blue catfish) and their reciprocal hybrids (CB, channel catfish female × blue catfish male, and BC, blue catfish female × channel catfish male) in tanks and other smaller culturing units (Figure 1A) (Dunham et al., 1987; Dunham et al., 1990; Argue et al., 2014; Wang et al., 2022a). In this study, we measured the body weight at 3 weeks and 10.8 months of age for N = 20 fish of each of the four genetic types (CC, BB, CB, and BC). No significant difference was observed between channel catfish and blue catfish at 3 weeks (*p* > 0.05; Figure 1B), but channel catfish were significantly heavier at 10.8 months than blue catfish (30.3 g vs 11.6g, *p* < 0.001; Figure 1C and Data S1). The difference in body weight suggests that the growth rate of channel catfish is approximately three times higher than that of blue catfish, which is consistent with previous studies (Wang et al., 2022c).

### Transcriptome-wide expression profiling revealed significant differences in gene expression in the liver, mucus, and intestine between channel catfish and blue catfish

To investigate gene expression differences in important organs between channel catfish and blue catfish, the heart, liver, intestine, mucus, and muscle were selected for transcriptome analysis at 10.8 months of age (Figure 1D and Supplementary Table S1). In total, 697 million 150-bp reads (209.2 Gbp of sequences) were generated (Supplementary Table S2). On average, 73% of RNA-seq reads were uniquely mapped to the blue catfish reference genome (Wang et al., 2022b). To investigate the tissue-specific gene expression profiles in both species, a multi-dimensional scaling (MDS) plot was generated using normalized gene counts and the transcriptomic profiles clustered together by tissue in the first two dimensions (Figure 1E). Overall, tissues exhibit a higher degree of resemblance than species, reflecting functional similarities among individual tissues. Dimension 1 separated skeletal muscle and heart from the remaining tissues, which is consistent with the fact that they are derived from mesoderm (Figure 1E). The skeletal muscle and heart tissues from BB and CC are intermingled, whereas liver, intestine, and skin mucus samples from the two species are well separated (Figure 1E). The results suggest that skeletal muscle and heart muscle are more functionally conserved than other tissues. Notably, skin mucus and intestine transcriptomic profiles are more similar within species. Mucus-secreting cells are also present in the intestine, and both organs are in contact with the microbiota (gut and skin microbiota).

### Detection of differentially expressed genes between channel catfish and blue catfish in five organs

**FIGURE 2:**
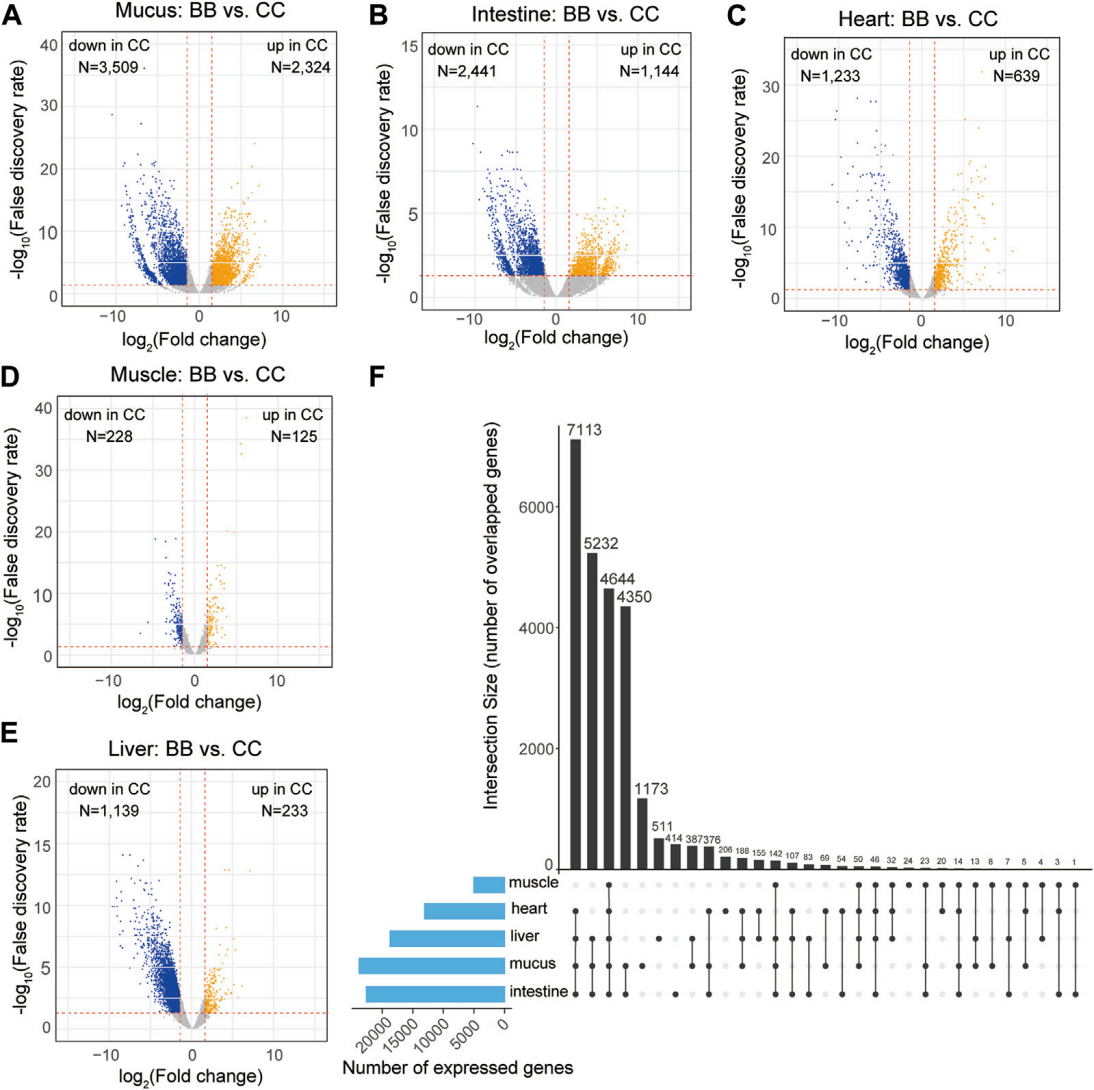
Transcriptome-wide differentially expressed genes (DEGs) in five organs between channel catfish and blue catfish. Volcano plots of pairwise comparisons in the mucus **(A)**, intestine **(B)**, heart **(C)**, muscle **(D)**, and liver **(E)**. DEGs with a false discovery rate (FDR) < 0.05 are highlighted. The *x*-axis stands for log2 (fold change), and the *y*-axis represents −log_10_(FDR). The vertical lines indicate |log2FoldChange| = 1.5. **(F)** Upset plot showing the intersection of expressed gene sets across five organs between channel catfish and blue catfish.

The expression levels of each gene were determined using the reads per kilobase of transcript per million mapped reads (RPKM). To identify DEGs, a pairwise analysis of differential gene expression was conducted between channel catfish and blue catfish (Data S2). Among these five organs, the mucus exhibited the largest number of DEGs, with 2,324 upregulated and 3,509 downregulated DEGs in channel catfish compared to blue catfish (Figure 2A). The intestine and heart also had more than 2,000 DEGs between channel catfish and blue catfish (Figures 2B,C). In contrast, muscle displayed the lowest number of DEGs, with only 125 upregulated DEGs and 228 downregulated DEGs (Figure 2D), suggesting functional conservation between species. There were ∼1,400 DEGs identified in the liver samples (Figure 2E).

To compare the overall expression profiles among five organs, the numbers of expressed genes (RPKM >2) in each organ were investigated (Figure 2F). The mucus transcriptome had the largest number of expressed genes (>20,000 genes), whereas the muscle had only 5,036 genes detected, indicating considerable variation among organs. A total of 4,644 genes were found to be shared among all five organs (Figure 2F), accounting for ∼90% of the muscle transcriptome or ∼25% of the mucus transcriptome. The liver, heart, intestine, and mucus exhibited the largest overlapping gene set, with 7,113 expressed genes common to these organs. More than 4,000 genes were expressed exclusively in the mucus and intestine, which is consistent with the similar gene expression profiles depicted in the MDS plot (Figure 1E). Regarding genes that were only expressed within an individual organ, mucus had the largest number of organ-specific genes (1,173), while muscle tissue had the lowest number, with only 24 such genes identified (Figure 2F).

### Stress response, immune activity, and metabolic pathways are enriched among DEGs

**FIGURE 3:**
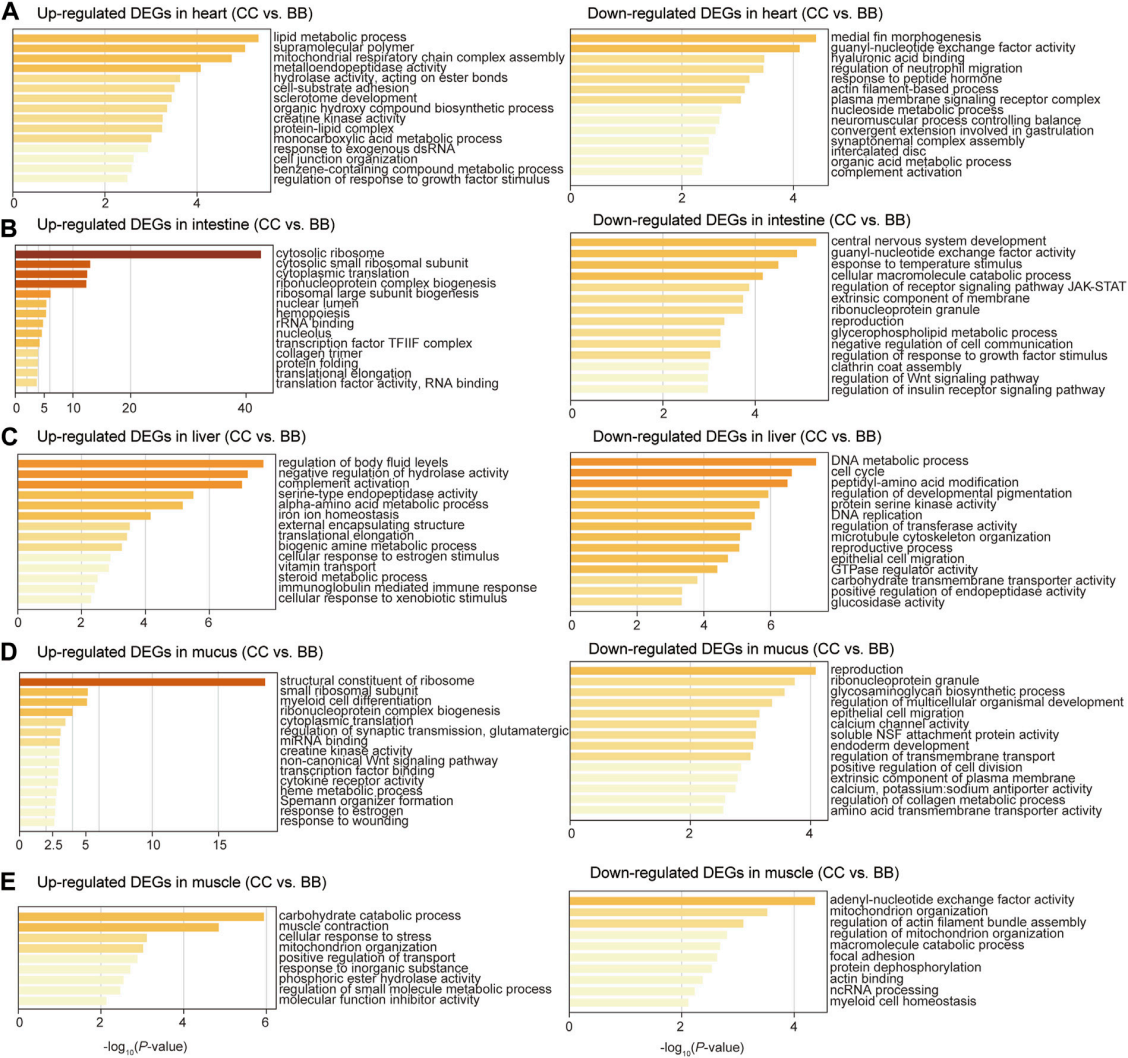
Gene ontology enrichment analysis of differentially expressed genes between channel catfish and blue catfish. Enrichment scores (*x*-axis) were determined by −log_10_(*p*-value) for significantly enriched terms of upregulated and downregulated genes in channel catfish in the heart **(A)**, intestine **(B)**, liver **(C)**, mucus **(D)**, and muscle **(E)**.

To identify the biological function of DEGs in each tissue, gene ontology (GO) enrichment analyses were performed comparing blue catfish and channel catfish. The upregulated DEGs were defined as genes with higher expression levels in channel catfish than those in blue catfish. In the heart, upregulated DEGs were significantly enriched in the lipid metabolic process (*p* < 10^−5^), while downregulated DEGs clustered in immunity-related terms such as regulation of neutrophil migration and complement activation (Figure 3A). In the intestine, the most significant function term was “cytosolic ribosome” in the upregulated DEGs (*p* < 10^−40^; Figure 3B). In contrast, the downregulated DEGs were mainly enriched in response to temperature stimulus (*p* < 10^−4^), which is associated with the stress response process (Figure 3B). In the liver, upregulated DEGs were primarily enriched in the cellular lipid metabolic process (*p* < 0.001; Figure 3C). Interestingly, downregulated DEGs were also clustered in metabolic terms such as carboxylic acid metabolic process, NADP metabolic process, and cellular lipid metabolic process (Figure 3C). In mucus, the enrichment analysis revealed that upregulated DEGs were significantly associated with the structural constituents of ribosomes (*p* < 10^−17^), creatine kinase activity, cytokine receptor activity, and response to wounding (Figure 3D). For muscle tissues, the top three most enriched terms among upregulated DEGs were carbohydrate catabolic process, muscle contraction, and cellular response to stress (Figure 3E).

### Tissue-specific genes in channel catfish and blue catfish

**FIGURE 4:**
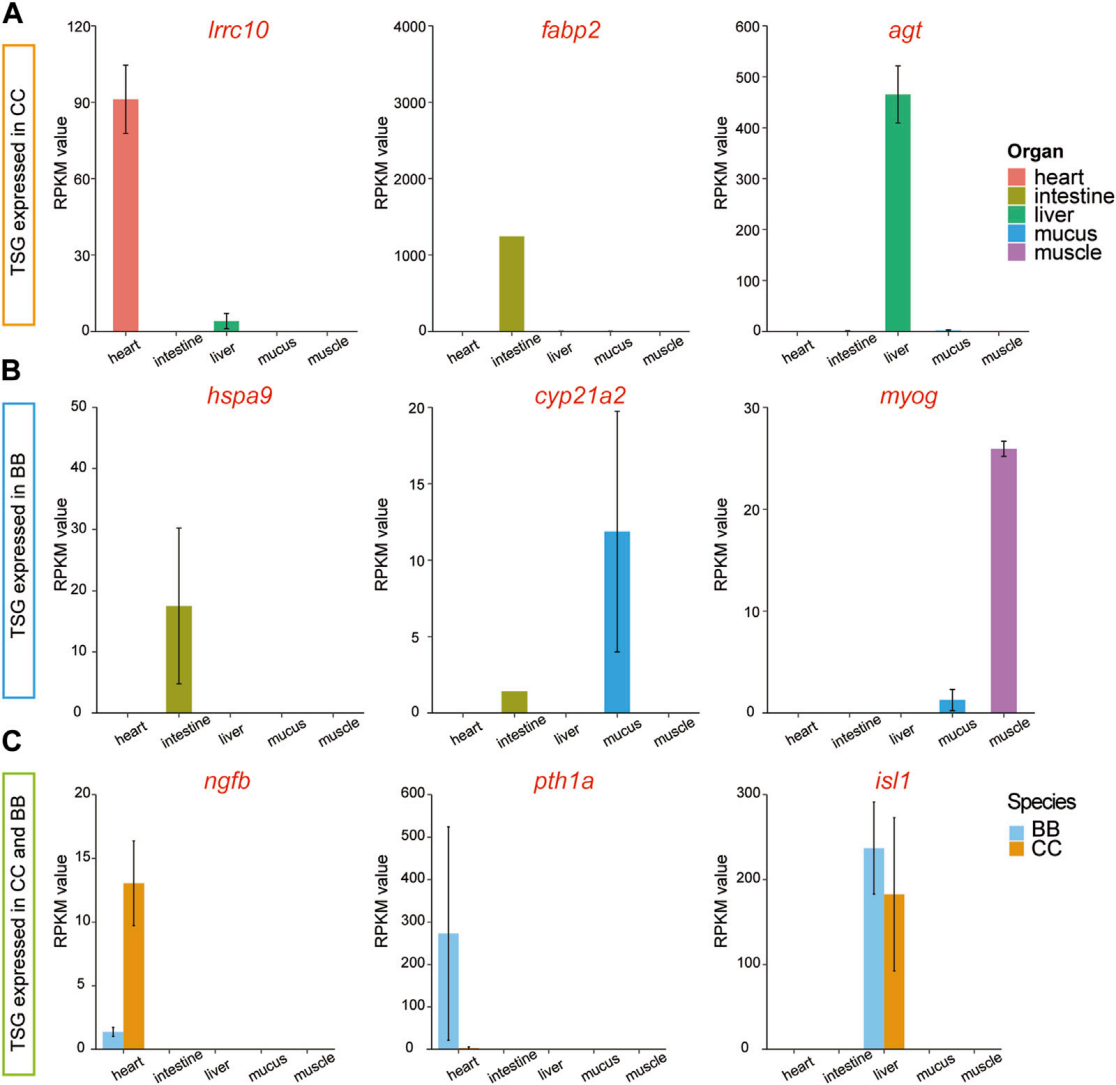
Tissue-specific and species-specific gene candidates in channel catfish and blue catfish at 10.8 months of age. Barplots of RNA-seq reads per kilobase of transcript per million mapped reads (RPKM) values. **(A)** Tissue-specific genes (*lrrc10*, *fabp2,* and *agt*) found only in channel catfish. **(B)** Tissue-specific genes (*hspa9*, *cyp21a2,* and *myog*) found only in blue catfish. **(C)** Tissue-specific genes (*ngfb*, *pth1a*, and *isl1*) found in channel catfish and blue catfish.To graphics: This image should be moved to figure 4

To examine the diversity of expression patterns among tissues, we utilized a tissue specificity index (τ value) to quantify the specificity of the gene profile (Data S3). In this study, a gene with a τ value greater than 0.9 was classified as a tissue-specific gene (TSG). Thus, TSGs were identified across the five tissues in two species. Among the different tissues in channel catfish, the number of TSGs ranged from 33 in muscle tissue to 1,872 in mucus tissue (Supplementary Table S3 and Supplementary Data S4). The distribution of TSG across tissues in blue catfish followed a similar pattern. Among the tissue-specific genes, three highly expressed genes, *irrc10*, *fabp2*, and *agt*, were exclusively detected in channel catfish (Figure 4A). Three different genes, *hspa9*, *cyp21a2*, and *myog*, were identified only in blue catfish. It is worth noting that some tissue-specific genes were expressed in both species, including *ngfb*, *pth1a*, and *isl1* (Figure 4C).

To confirm tissue-specific expression identified from RNA-seq data using an independent approach, qRT-PCR was performed to determine the relative expression levels of selected genes (see Materials and Methods). The relative expression value of *fabp2* was significantly higher in the intestine of channel catfish than in other tissues (*p* < 0.05; Supplementary Figure S1). No *fabp2* expression was detected in blue catfish, indicating that *fabp2* is intestine-biased in channel catfish only. *Cyp21a2* was highly expressed in both the intestine and mucus of blue catfish, while little to no expression was detected in channel catfish (Supplementary Figure S2). *Pth1a* was exclusively expressed in the heart in both species, but its expression level in the blue catfish was over 100 times higher than in the channel catfish (Supplementary Figure S3). The relative expression values of all three genes are consistent with the expression pattern identified from RNA-seq data.

## Discussion

### Tissue-specific transcriptomes in blue catfish and channel catfish

In this study, transcriptomes from five major tissues/organs provide a genomic resource for investigating the transcriptomic differences between two economically important catfish species. Given the variations observed in growth performance and disease resistance between blue catfish and channel catfish, there is a strong interest in understanding local adaptation, genome evolution, and the genetic basis underlying these traits. Over the past decade, expressed sequence tag (EST) sequencing (Li et al., 2007), single nucleotide polymorphisms (SNPs) information (Liu et al., 2011), and full-length cDNAs identification (Chen et al., 2010) have been characterized in blue catfish and channel catfish. Although swimbladder RNA-seq data were reported to investigate the differences in chamber formation between blue catfish and channel catfish (Yang et al., 2018), the transcriptomic divergence study across multiple tissues between these two species is still limited.

Transcriptomes are most commonly used in fish to characterize molecular physiology and identify genes that respond to or ameliorate environmental stresses (Basu et al., 2002; Cossins and Crawford, 2005). Gene expression regulation shows considerable variation among different organs, individuals, and species (Hsieh et al., 2003). All tissue-specific transcriptomes used in this study were from peripheral tissues, including the heart, intestine, liver, mucus, and muscle. Overall, the peripheral tissue transcriptomes separated into distinct clusters on the MDS plot, suggesting a divergence of gene expression patterns. The utilization of peripheral tissue transcriptomes in catfish research can contribute to the understanding of catfish biology and create opportunities for further investigations in various fields, including metabolism, immune responses, development, stress response, and physiology.

### The growth advantage in channel catfish may be associated with metabolic regulation and tissue development

Cardiac tissue plays a vital role in fish physiology as it is responsible for pumping oxygenated blood throughout the fish’s body and delivering essential nutrients and hormones to various organs and tissues. Cardiac transcriptome analyses have been very effective in discovering candidate genes in studies of cardiac toxicity, response to hypoxia, and cardiac disease in various fish species (Shih et al., 2015; Saetan et al., 2021; Xiao et al., 2021). Although no treatment was administered in the present study, two heart tissue-specific genes were identified through a comparison of gene profiles among tissues, including *lrrc10* and *pth1a* (Figure 3). *Irrc10*, a highly conserved gene unique to the heart, is implicated in embryonic development and tissue differentiation processes. *Lrrc10* was reported as a cardiac-specific factor (*Serdin1*) in mice that is essential for heart development (Adameyko et al., 2005; Manuylov et al., 2008). In zebrafish, the *Lrrc10* morphants exhibited cardiac functional defects, as evidenced by a decrease in ejection fraction and cardiac output (Kim et al., 2007). The *pth1a* gene encodes a protein called parathyroid hormone receptor 1 (PTH1 receptor), which plays a crucial role in the regulation of calcium and phosphate homeostasis. This is consistent with the downregulated DEGs enriched in response to peptide hormones (Figure 3A). It was also shown to play a role in bone remodeling, which involves the continuous breakdown and formation of bone tissue, in zebrafish (Aceto et al., 2015).

Fish intestine serves several important functions related to digestion, nutrient absorption, body fluid balance, and immune defense, which are critical for growth and disease-resistance phenotypes. A dramatically upregulated intestine-specific gene in channel catfish, *fabp2* (fatty acid-binding protein 2; Figure 4A), encodes an intestinal fatty acid-binding protein (I-FABP). Fatty acid-binding proteins (FABPs) play a crucial role in the transcriptional regulation of genes associated with lipid metabolism, which can significantly impact fat deposition in animals (Venkatachalam et al., 2018). *Fabp2* was initially identified in mammals and is expressed exclusively in the intestine (Gajda and Storch, 2015). It has also been reported in fish species with variable expression patterns (Sharma et al., 2004; Venkatachalam et al., 2017). Numerous studies have provided evidence that the expression of FABP in fish is regulated by nutritional factors (Venold et al., 2013; Xu et al., 2017). Starvation stress affects the expression level of FABP in various fish species, typically leading to downregulation in response to prolonged periods of starvation (Kaitetzidou et al., 2015; Ölmez et al., 2015). Therefore, alterations in FABP levels and gene expression can serve as indicators of lipid accumulation. During the dissection process in the present study, a substantial presence of white adipose tissues was notably observed in channel catfish, indicating that channel catfish tend to accumulate a greater amount of energy sources than blue catfish at the 10.8-month developmental stage.

Skeletal muscle constitutes the major portion of the fish trunk, comprising approximately 40%–60% of the total body weight (Xu et al., 2019). Fish muscle performs a variety of physiological functions associated with locomotion, movement, and metabolism. The regulation of muscle fiber development and growth is maintained by myogenic regulatory factor (MRF) genes, including *myod*, *myf5*, *myog*, and *mrf4*. Among these genes, *myog* is a crucial member of the myogenic regulator family, responsible for governing the differentiation of mesodermal cells into myoblasts, which subsequently form the muscle fibers. Notably, *myog* is the only gene among the MRFs expressed in all skeletal muscle cell lines (Hasty et al., 1993). It was also reported that the MRF genes are regulated by the GH-insulin-like growth factor (IGF) axis (Fuentes et al., 2013). Gene silencing and knockout of the myostatin gene have been found to promote somatic growth in many fish species, such as zebrafish (Gao et al., 2016), medaka (Chiang et al., 2016), and channel catfish (Khalil et al., 2017). In the present study, the highly expressed *myog* gene in the muscle of channel catfish may contribute to the difference in growth performance between blue catfish and channel catfish in a tank environment (Figure 4B).

### Difference in stress response and immune activity between blue catfish and channel catfish

Mucus provides the mucosal barrier as the first line of defense against pathogens. The secretion patterns of mucus not only influence the rates of bacterial shedding but also play important roles in the production of enzymes, antimicrobial peptides, and secreted immunoglobulins (Xu et al., 2013). Comparing the mucus transcriptome between blue catfish and channel catfish revealed a significant number of DEGs, indicating potential differences in immune response mechanisms. Specifically, the upregulated DEGs in mucus were enriched with gene ontology terms related to cytokine receptor activity (GO: 0004896) and response to wounding (GO: 0009611), suggesting unique immune patterns in channel catfish (Figure 3D). Blue catfish also have their own set of GO terms for stress response. For example, a mucus-specific gene, *cyp21a2* was highly expressed in blue catfish compared to channel catfish (Figure 4B). It encodes the cytochrome P450 enzyme 21-hydroxylase, which plays a crucial role in catalyzing a key step in the biosynthesis of glucocorticoids (such as cortisol) and mineralocorticoids (Miller and Auchus, 2011). In fish, cortisol serves as the primary circulating glucocorticoid, with effects mediated through the glucocorticoid receptor (GR) (Faught and Vijayan, 2016). The primary function of *cyp21a2* is to facilitate the production of cortisol, which is involved in regulating metabolism, immune responses, stress response, and maintaining homeostasis in the body. In zebrafish, *cyp21a2* knockout induced a reduction in cortisol levels (Eachus et al., 2017). As a common stress indicator, the higher cortisol levels in blue catfish may indicate that this species is more sensitive to environmental and psychological stress. Further study needs to be conducted at the physiological level to confirm this. In addition to glucocorticoids, *cyp21a2* is also responsible for the production of mineralocorticoids, primarily aldosterone. Aldosterone is involved in regulating sodium and potassium balance, blood pressure, and fluid balance in the body.

The liver plays an important role in the metabolism, detoxification, nutrient storage, synthesis of blood proteins, and immune function of fish. The *agt* gene, also known as angiotensinogen, encodes the angiotensinogen protein. Angiotensinogen is a precursor protein that plays a significant role in the renin-angiotensin system (RAS) (Nishimura, 2004), a hormonal cascade involved in regulating blood pressure, fluid balance, and sodium homeostasis. The *agt* gene is also likely to be involved in the immune response, as a previous study indicated that the expression level of *agt* significantly increased after bacterial infection in ayu (Chen et al., 2008).

Heat tolerance is a critical trait in aquaculture species (Tan et al., 2019). Heat shock proteins (HSPs) belong to a superfamily of proteins that are triggered by various stressors, including physical, chemical, and biological factors, such as high temperature, hypoxia, infection, and toxins (Kregel, 2002). HSP70 is a widely recognized stress protein in aquatic organisms, playing a crucial role in stress responses, including thermotolerance (Bertotto et al., 2011), and also participating in the regulation of the immune system (Tsan and Gao, 2009). In the present study, *hspa9*, as a member of the heat shock protein 70 (HSP70) family, was found to be highly expressed in blue catfish intestine tissue compared to channel catfish (Figure 4B), indicating potential stress susceptibility in blue catfish. In Japanese flounder, the *hspa9* was identified with a high level of expression in the transcriptome after infection with *Edwardsiella tarda*.

The sensory and neural systems enable fish species to perceive the world around them and respond appropriately to the environment (Borghezan et al., 2021). The *ngfb* gene encodes the nerve growth factor beta (NGFβ) protein, which plays a vital role in the development and survival of nerve cells, particularly sensory neurons responsible for transmitting pain, temperature, and touch sensations. A significant difference in *ngfb* expression levels was observed in this study between channel catfish and blue catfish (Figure 4C), which may impact their function. It was found that *ngfb* was downregulated in the hippocampal neurons induced by lipopolysaccharides (LPS) (Fang et al., 2020). Collectively, divergence in multiple organs may contribute to the differences in stress response and immune activity between channel catfish and blue catfish.

## Data Availability

The raw RNA-seq datasets and gene read counts for this study can be found in the NCBI GEO (Gene Expression Omnibus) databases under the accession number GSE247918.
