# Supplementary material for: Transcriptome analysis revealed potential mechanisms of channel catfish growth advantage over blue catfish in a tank culture environment
Source: Front Genet. 2024 Apr 29;15:1341555. doi: 10.3389/fgene.2024.1341555 (PMC11089159; doi:10.3389/fgene.2024.1341555)
Supplement: Supplementary file 1 [file DataSheet2.PDF]

Table S1. List of organ samples used in this study.

Sample_ID	Species	Tissue/organ	Age	Replicate
BB_Tnk_10m1_hart	Ictalurus furcatus	heart	10.8 month	Rep1
BB_Tnk_10m1_inte	Ictalurus furcatus	intestine	10.8 month	Rep1
BB_Tnk_10m1_live	Ictalurus furcatus	liver	10.8 month	Rep1
BB_Tnk_10m1_mucu	Ictalurus furcatus	mucus	10.8 month	Rep1
BB_Tnk_10m1_musl	Ictalurus furcatus	muscle	10.8 month	Rep1
BB_Tnk_10m2_hart	Ictalurus furcatus	heart	10.8 month	Rep2
BB_Tnk_10m2_inte	Ictalurus furcatus	intestine	10.8 month	Rep2
BB_Tnk_10m2_live	Ictalurus furcatus	liver	10.8 month	Rep2
BB_Tnk_10m2_mucu	Ictalurus furcatus	mucus	10.8 month	Rep2
BB_Tnk_10m2_musl	Ictalurus furcatus	muscle	10.8 month	Rep2
BB_Tnk_10m3_hart	Ictalurus furcatus	heart	10.8 month	Rep3
BB_Tnk_10m3_inte	Ictalurus furcatus	intestine	10.8 month	Rep3
BB_Tnk_10m3_live	Ictalurus furcatus	liver	10.8 month	Rep3
BB_Tnk_10m3_mucu	Ictalurus furcatus	mucus	10.8 month	Rep3
BB_Tnk_10m3_mus	Ictalurus furcatus	muscle	10.8 month	Rep3
CC_Tnk_10m1_hart	Ictalurus punctatus	heart	10.8 month	Rep1
CC_Tnk_10m1_inte	Ictalurus punctatus	intestine	10.8 month	Rep1
CC_Tnk_10m1_live	Ictalurus punctatus	liver	10.8 month	Rep1
CC_Tnk_10m1_mucu	Ictalurus punctatus	mucus	10.8 month	Rep1
CC_Tnk_10m1_musl	Ictalurus punctatus	muscle	10.8 month	Rep1
CC_Tnk_10m2_hart	Ictalurus punctatus	heart	10.8 month	Rep2
CC_Tnk_10m2_inte	Ictalurus punctatus	intestine	10.8 month	Rep2
CC_Tnk_10m2_live	Ictalurus punctatus	liver	10.8 month	Rep2
CC_Tnk_10m2_mucu	Ictalurus punctatus	mucus	10.8 month	Rep2
CC_Tnk_10m2_musl	Ictalurus punctatus	muscle	10.8 month	Rep2
CC_Tnk_10m3_hart	Ictalurus punctatus	heart	10.8 month	Rep3
CC_Tnk_10m3_inte	Ictalurus punctatus	intestine	10.8 month	Rep3
CC_Tnk_10m3_live	Ictalurus punctatus	liver	10.8 month	Rep3
CC_Tnk_10m3_mucu	Ictalurus punctatus	mucus	10.8 month	Rep3
CC_Tnk_10m3_musl	Ictalurus punctatus	muscle	10.8 month	Rep3

Table S2. RNA sequencing yield and mapping percentages.

Sample_ID	Total number of reads	Number of filtered reads	Uniquely mapping percentage
BB_Tnk_10m1_hart	23350062	22831165	85.4%
BB_Tnk_10m1_inte	11654912	11365616	44.3%
BB_Tnk_10m1_live	23771260	22622666	93.8%
BB_Tnk_10m1_mucu	12544018	11993844	76.2%
BB_Tnk_10m1_musl	25223739	24146788	89.8%
BB_Tnk_10m2_hart	20560696	20157247	83.9%
BB_Tnk_10m2_inte	13273693	12855675	50.6%
BB_Tnk_10m2_live	25707722	24608300	93.4%
BB_Tnk_10m2_mucu	11595604	11181368	76.8%
BB_Tnk_10m2_musl	22024998	21065178	88.5%
BB_Tnk_10m3_hart	16809535	16415798	79.0%
BB_Tnk_10m3_inte	11824226	11407636	67.3%
BB_Tnk_10m3_live	21849349	21173198	94.3%
BB_Tnk_10m3_mucu	12250040	11798444	77.6%
BB_Tnk_10m3_mus	19692097	18499419	88.3%
CC_Tnk_10m1_hart	35051088	34245424	72.3%
CC_Tnk_10m1_inte	13168015	12892282	40.9%
CC_Tnk_10m1_live	25832403	24733844	92.1%
CC_Tnk_10m1_mucu	17996558	17579631	56.0%
CC_Tnk_10m1_musl	15036823	14103186	83.6%
CC_Tnk_10m2_hart	21697501	21198676	72.0%
CC_Tnk_10m2_inte	35971620	35226724	33.3%
CC_Tnk_10m2_live	35622225	34783914	48.9%
CC_Tnk_10m2_mucu	10197289	9748299	65.5%
CC_Tnk_10m2_musl	13358157	12568518	86.3%
CC_Tnk_10m3_hart	24478985	23893386	74.5%
CC_Tnk_10m3_inte	17656989	17183823	44.9%
CC_Tnk_10m3_live	14424191	13879208	92.6%
CC_Tnk_10m3_mucu	11615211	11338863	47.0%
CC_Tnk_10m3_musl	16685604	15670589	82.6%

Table S3. Number of tissue-specific gene (TSG) expressed in channel catfish and blue catfish.

Number of TSGs	Heart	Intestine	Liver	Mucus	Muscle
Blue catfish	266	635	613	1640	37
Channel catfish	252	928	716	1872	33

The cutoff of τ index value is 0.9.

Table S4. Primer sequences for qRT-PCR validation of tissue-specific genes in blue catfish and channel catfish.

Gene name	Species	Primes sequences (5'-3')	Product size (bp)
cyp21a2-F	blue catfish	CCTGTTTGAGGTATAGCGCTT	171
cyp21a2-R	blue catfish	TTTGCAGGAAGACCGCATTCA	
pth1a-F	blue catfish	CATAAGGACAGACAGCAGACC	242
pth1a-R	blue catfish	TATCTCCAGTGTTTCCACC	
fabp2-F	channel catfish	GATGATCATGTCGGTGCGT	178
fabp2-R	channel catfish	TCGACATGGACTGGACCAC	
gapdh-F	blue/channel	AGACTTCAATGGAGATACTCA	111
gapdh-R	blue/channel	CTGTAGCCAAACTCGTTG	
