# Supplementary material for: Transcriptome analysis revealed potential mechanisms of channel catfish growth advantage over blue catfish in a tank culture environment
Source: Front Genet. 2024 Apr 29;15:1341555. doi: 10.3389/fgene.2024.1341555 (PMC11089159; doi:10.3389/fgene.2024.1341555)

Figure S1. Quantitative reverse transcription PCR validation of intestine-specific gene *fabp2* in channel catfish.

Barplots of qRT-PCR relative expression value in 10.8-month heart, intestine, liver, mucus, and muscle of channel catfish with three biological replicates. The data is presented as mean+SEM.

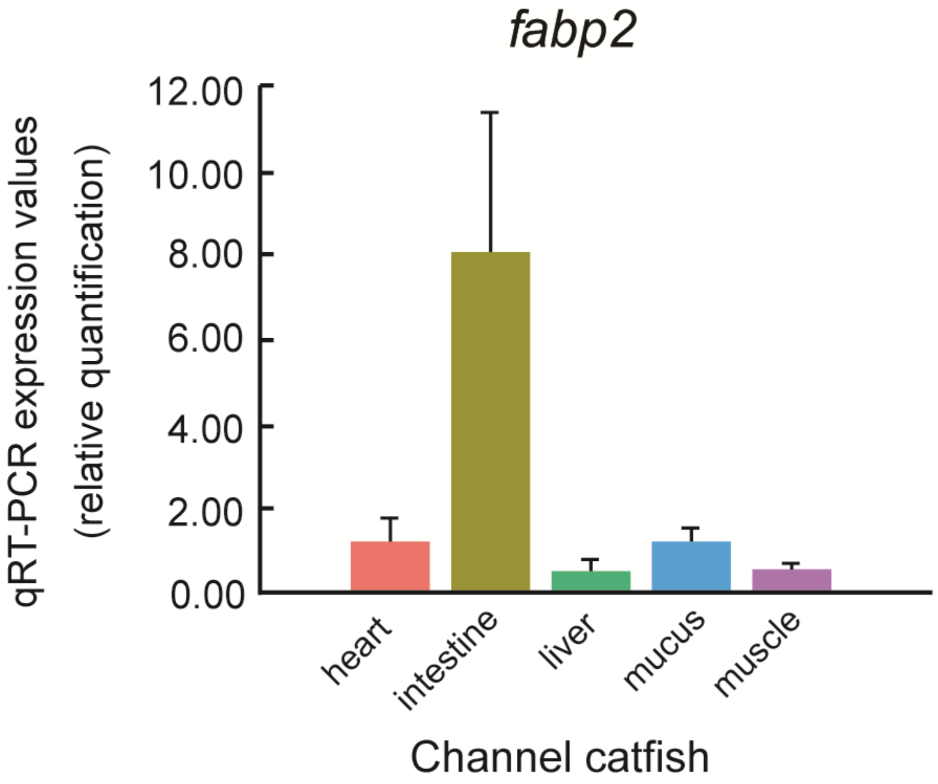

Figure S2. Quantitative reverse transcription PCR validation of mucus-biased gene *cyp21a2* in blue catfish.

Barplots of qRT-PCR relative expression value in 10.8-month heart, intestine, liver, mucus, and muscle of blue catfish with three biological replicates. The data is presented as mean+SEM.

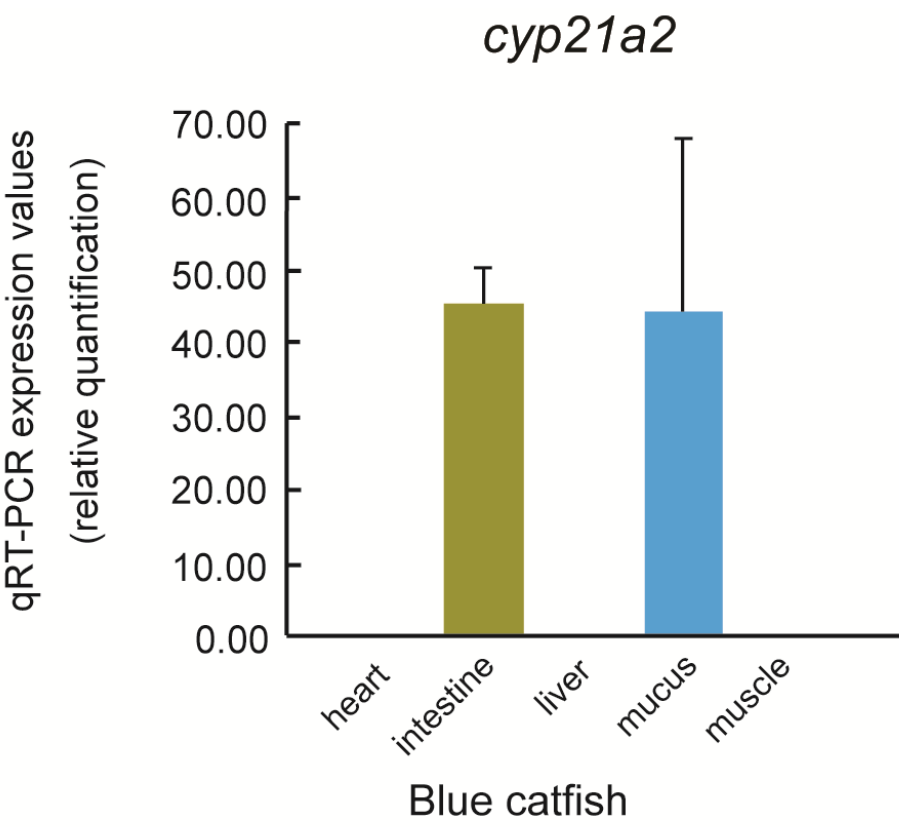

Figure S3. Quantitative reverse transcription PCR validation of heart-specific gene *pth1a* in blue catfish.

Barplots of qRT-PCR relative expression value in 10.8-month heart, intestine, liver, mucus, and muscle of channel catfish and blue catfish with three biological replicates per species. The data is presented as mean+SEM.

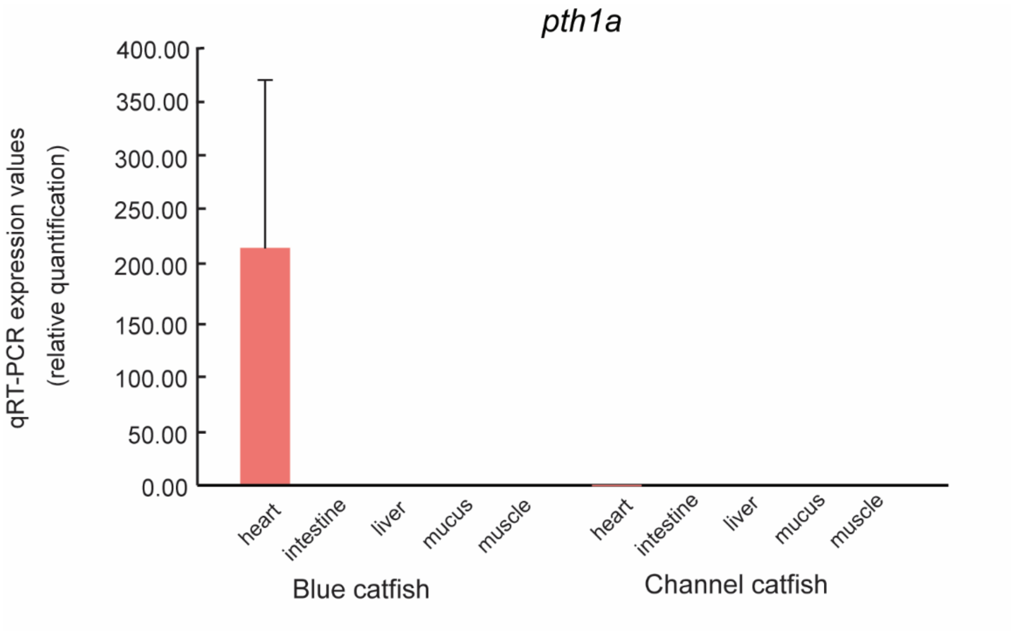

Supplement: Supplementary file 2 [file DataSheet1.PDF]
